# Preliminary Educational Requirement in Texas

**Published:** 1910-12

**Authors:** 


### Preliminary Educational Requirement in Texas.

The following communication has been received from Dr. M. E. Daniel, Honey Grove, Texas, Chairman of the College and Reciprocity Committee of the Texas State Board of Medical Examiners:

"This is to notify prospective medical students and all concerned, that before matriculation in the medical colleges of Texas is permissible, it is necessary to procure an entrance certificate from the State Board of Medical Examiners. This means that the authorities of the several medical colleges no longer pass on the literary credentials of applicants for matriculation. This function is now exercised by the State Board of Medical Examiners.

"To be eligible for examination or reciprocity, all Texas students matriculating in other States since 1908 must comply with this requirement. Non-residents desiring matriculation are required to comply with this regulation whether they expect to take the State Medical Board examination or not. Texas medical colleges are required to matriculate resident and non-resident students on equal terms.

"Residents and non-residents alike, who comply with the entrance requirements in such States as have reciprocal relations with Texas, will be accepted for matriculation, examination or reciprocity, provided literary credentials are acted on and credits determined by the State Medical Examining Board or other legally designated authority independent of medical college authorities or faculties.

"Residents and non-residents matriculating in States other than reciprocal States, and who expect future legalization in Texas, must comply with this regulation, but matriculants of such of these States as maintain and enforce an educational standard equal to our own, will be accepted. But those matriculating in States which maintain no fixed literary prerequisite, where the college faculties have the sole prerogative of passing on and determining the literary qualifications of medical students, will be barred unless they possess an entrance certificate issued by this board or from some other State having reciprocal relations with Texas. It is not sufficient to possess the necessary literary credential with the view of submitting it after graduation; the required entrance certificate must be procured before matriculation.

"Texas now reciprocates with the following States: Arkansas, Missouri, Illinois, Indiana, Iowa, Michigan, Kentucky, Maine, Nebraska, Minnesota, District of Columbia, West Virginia, Virginia, Maryland, Wisconsin, Vermont, North Dakota, Ohio, New Jersey, Kansas and Nevada.

"Full particulars can be had by addressing the Secretary of the Board, Dr. R. H. McLeod, Palestine, Texas."

---

"Sexual neurasthenia" in the male not infrequently depends on disease in the posterior urethra.—*American Journal of Surgery.*

